# Supplementary figures and images for: Highly diverse–Low abundance methanogenic communities in hypersaline microbial mats of Guerrero Negro B.C.S., assessed through microcosm experiments
Source: PLoS One. 2024 Oct 4;19(10):e0303004. doi: 10.1371/journal.pone.0303004 (PMC11451985; doi:10.1371/journal.pone.0303004)

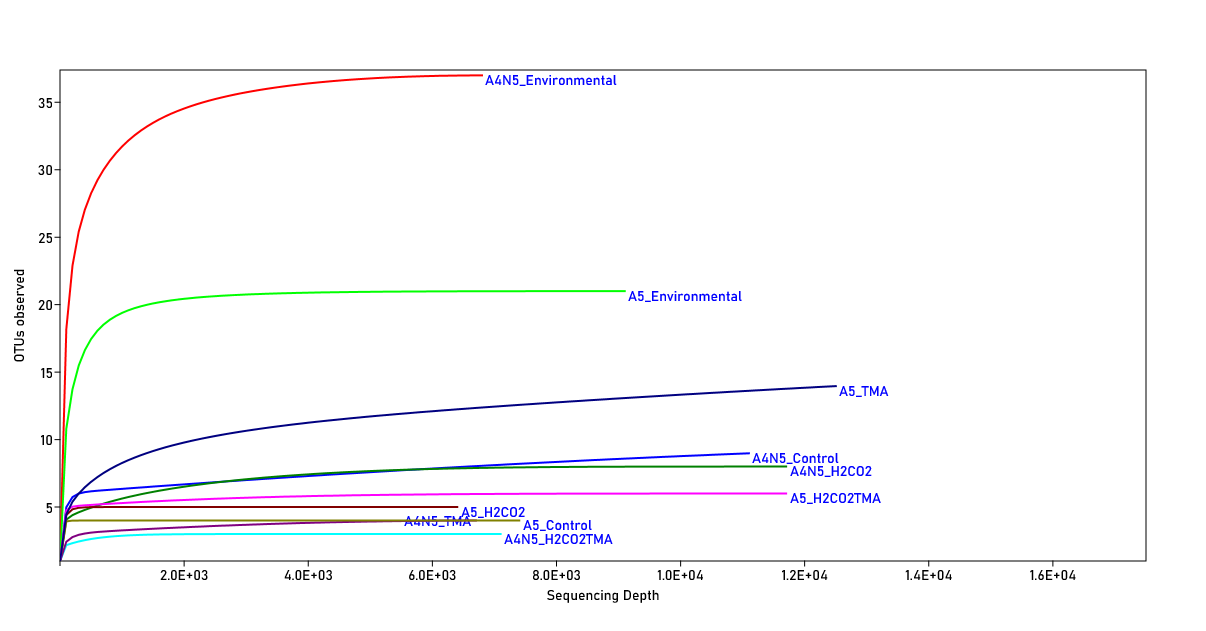

Supplement: S1 Fig — Rarefaction curves based on the arithmetic mean of OTUs observed in the microbial communities of both environmental samples and treatments (control and enrichments) from both sampling sites. The x axis indicates the size of the library, and the y axis represents the number of OTUs detected by each sample. (PNG) [file pone.0303004.s001.png]
